# The Serine Biosynthesis of *Paenibacillus polymyxa* WLY78 Is Regulated by the T-Box Riboswitch

**DOI:** 10.3390/ijms22063033

**Published:** 2021-03-16

**Authors:** Haowei Zhang, Qin Li, Yongbin Li, Sanfeng Chen

**Affiliations:** State Key Laboratory for Agrobiotechnology and College of Biological Sciences, China Agricultural University, Beijing 100094, China; zhanghw@cau.edu.cn (H.Z.); lqliqin1@126.com (Q.L.); ybli@cau.edu.cn (Y.L.)

**Keywords:** T-box riboswitch, *Paenibacillus polymyxa* WLY78, serine, *serA*

## Abstract

Serine is important for nearly all microorganisms in protein and downstream amino acids synthesis, however, the effect of serine on growth and nitrogen fixation was not completely clear in many bacteria, besides, the regulatory mode of serine remains to be fully established. In this study, we demonstrated that L-serine is essential for growth and nitrogen fixation of *Paenibacillus polymyxa* WLY78, but high concentrations of L-serine inhibit growth, nitrogenase activity, and *nifH* expression. Then, we revealed that expression of the *serA* whose gene product catalyzes the first reaction in the serine biosynthetic pathway is regulated by the T-box riboswitch regulatory system. The 508 bp mRNA leader region upstream of the *serA* coding region contains a 280 bp T-box riboswitch. The secondary structure of the T-box riboswitch with several conserved features: three stem-loop structures, a 14-bp T-box sequence, and an intrinsic transcriptional terminator, is predicted. Mutation and the transcriptional leader-*lacZ* fusions experiments revealed that the specifier codon of serine is AGC (complementary to the anticodon sequence of tRNA^ser^). qRT-PCR showed that transcription of *serA* is induced by serine starvation, whereas deletion of the specifier codon resulted in nearly no expression of *serA*. Deletion of the terminator sequence or mutation of the continuous seven T following the terminator led to constitutive expression of *serA*. The data indicated that the T-box riboswitch, a noncoding RNA segment in the leader region, regulates expression of *serA* by a transcription antitermination mechanism.

## 1. Introduction

Riboswitches, one type of non-coding RNAs, are genetic regulation elements commonly located in the 5′-untranslated regions (5′-UTRs) of mRNAs [1,2,3]. Many bacteria use riboswitches to detect a variety of metabolites and ions to regulate gene expression. Riboswitches consist of two functional domains, the ligand-binding aptamer domain and the downstream adjoining expression platform [4,5]. The aptamer domain is able to bind a small molecule with high selectivity and affinity, such as amino acids, nucleobases, vitamin cofactors, and secondary messenger molecules [6,7,8,9,10,11]. The binding of a ligand to the aptamer domain will cause conformation changes that are transduced into the expression platform domain which finally results in expression regulation of downstream genes [4]. To date, more than 25 riboswitch classes that respond to various signals have been identified and characterized [12]. For example, the expression of methionine and cysteine biosynthesis genes is controlled by the S-box riboswitch in which S-adenosylmethionine (SAM) is the molecular effector sensed by the S-box leader RNAs during transcription [13,14]. 

The T-box riboswitches commonly modulate the expression of many genes encoding aminoacyl-tRNA synthetase, as well as genes encoding selected amino acid synthetase and transporter in Gram-positive bacteria [15,16,17,18]. A T-box RNA consists of a segment of leader RNA with conserved features. One feature includes a T-box sequence consisting of a conserved 14-nucleotide sequence (5′-AGGGUGGNACCGCG-3′) and another feature includes an element (a G+C-rich helix followed by a run of U residues) that serves as an intrinsic transcriptional terminator [16,19,20,21,22]. The T-box sequence is predicted to form the 5′ side of an alternate, less stable antiterminator structure that competes with the formation of the more stable terminator helix [15]. In addition to the segments that can form the terminator/antiterminator elements, the major structures formed within the T-box RNA are designated stem I, stem II, the stem IIA/stem IIB pseudoknot, and stem III [19,23,24]. Stem I contains the specifier sequence (complementary to the anticodon sequence of tRNA) that selectively pairs with a cognate tRNA [25]. The specifier sequence binds charged tRNA or uncharged tRNA in response to amino acid starvation. Binding of a specific uncharged tRNA stabilizes the antiterminator, prevents the formation of the terminator helix, and thus promotes expression of the downstream coding sequence. Whereas, binding of charged tRNA promotes the formation of the terminator helix indirectly and thus represses expression of the downstream genes [15,26].

The amino acid L-serine (Ser) is required for protein synthesis. Serine is also a chassis material for the biosynthesis of glycine, cysteine, and tryptophan. Glycine, in turn, is a precursor for purines and heme [27,28,29,30,31,32,33]. The first reaction in the serine biosynthetic pathway is the conversion of 3-phosphoglycerate to 3-phosphohydroxypyruvat catalyzed by 3-phosphoglycerate dehydrogenase encoded by the *serA* gene [13]. The regulation mechanism of *serA* in most members of the Firmicutes is unknown, although a T-box sequence is found in the *serA* regulatory region in *Bacillus clausii*, *Bacillus halodurans*, *Clostridiium acetobutylicum*, *Clostridiium tetani*, *Clostridium kluyveri,* and *Clostridium novyi* [19,34]. According to the RegPrecise database, in several *Bacillus* spp. (including *B. subtilis*) and other Firmicutes., *serA* is also regulated by the global transcriptional regulator CodY which is a sensor of nutrient availability [35], suggesting that regulation of *serA* is important to bridge the pathways between glycolysis and serine biosynthesis.

In this study, we examined the effects of L-serine on growth, nitrogenase activity, and *nifH* expression of *P. polymyxa* WLY78 which is a nitrogen-fixing bacterium and has great potential usage as a biofertilizer in agriculture. Then, we searched the genome sequence of the *P. polymyxa* WLY78 and found that a *serA* and a *serC* that are predicted to catalyze the first and the second reactions in the serine biosynthesis pathway are distributed separately. 5′-RACE (5′-Rapid Amplification of cDNA End) showed that the leader region upstream of the *serA* coding region is 508 bp in length and there is a 280 bp T-box region with several conserved sequences in the leader region. Alternation analysis of the three codons of serine revealed that the specifier codon is AGC. qRT-PCR analysis of the four mutants carrying the mutations in the T-box region demonstrated that the specifier codon and termination structure are essentially required for regulation of the *serA* expression. Our study not only reveals the regulation mechanisms of serine biosynthesis in *P. polymyxa* WLY78, but also will provide assistance for understanding the relationship between amino acids and nitrogen fixation.

## 2. Results

### 2.1. Impacts of Serine Concentration on Growth, Nitrogenase Activity, and nifH Expression of P. polymyxa WLY78

**Figure 1:**
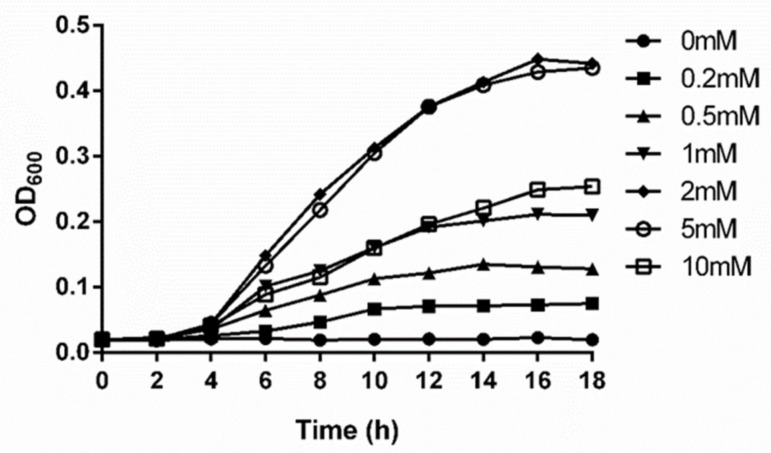
The growth rates of *P. polymyxa* WLY78 cultivated in minimal medium with L-serine as the sole nitrogen source. L-serine concentrations ranged from 0 to 10 mM. Results are representative of at least three independent experiments, mean values were calculated from three sets of independent experiments.

To test whether L-serine could be used as the sole nitrogen source for the growth of *P. polymyxa* WLY78, 0–10 mM L-serine was supplemented in medium lacking nitrogen source. As shown in Figure 1, *P. polymyxa* WLY78 did not grow in medium without L-serine. It grew well in presence of L-serine and showed the best growth in the presence of 2 mM L-serine (Figure 1), suggesting that L-serine can be used as the sole nitrogen source for the growth of *P. polymyxa* WLY78.

**Figure 2:**
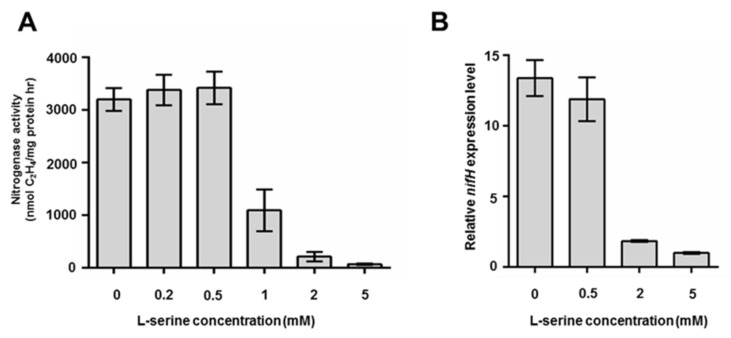
Nitrogenase activity and *nifH* expression of *P. polymyxa* WLY78 grown in different concentrations of serine. (**A**) The nitrogenase activity of *P. polymyxa* WLY78 grown in nitrogen-limited medium containing 0-5 mM L-serine. (**B**) The *nifH* expression level of *P. polymyxa* WLY78 grown in nitrogen-limited medium containing 0-5 mM L-serine determined by qRT-PCR. Results are representative of at least three independent experiments, mean values ± SDs were calculated from three sets of independent experiments.

Impacts of serine concentration on nitrogenase activities and *nifH* expression of *P. polymyxa* WLY78 were here investigated. *P. polymyxa* WLY78 showed high nitrogenase activities when it was grown in nitrogen-limited medium containing 0–0.5 mM of serine, but activities decreased as serine concentration increased (Figure 2A). qRT-PCR showed that the *nifH* of *P. polymyxa* WLY78 was transcribed in medium containing 0–0.5 mM L-serine, but it was not transcribed in medium containing 2 mM L-serine or 5 mM L-serine (Figure 2B), suggesting that 2 mM and more than 2 mM L-serine inhibited nif transcription. The data was in agreement with the nitrogenase activity observed above.

### 2.2. The serA Gene Is Essential for Growth of P. polymyxa WLY78 in Absence of Serine

We searched the genome of *P. polymyxa* WLY78 and found a *serA* gene was located between a *resDE* operon (coding for a two-component regulatory system) and a CBPB gene (coding for a CPBP family intramembrane metalloprotease). A *serC* gene is located elsewhere far from *serA* on the genome of *P. polymyxa* WLY78, but the *serB* gene is not found.

**Figure 3:**
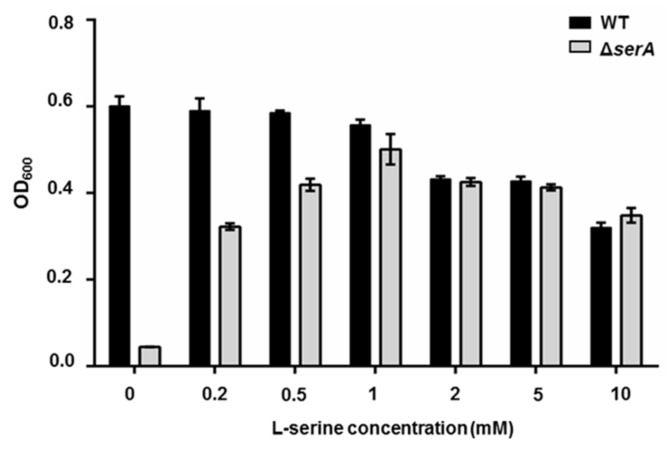
Comparison of the growth of *P. polymyxa* WLY78 and the Δ*serA* mutant in medium containing different concentrations of serine. The OD_600_ was measured at the stationary phase when growth reached maximum value. The medium contained NH_4_NO_3_ as the nitrogen source. Results are representative of at least three independent experiments, mean values ± SDs were calculated from three sets of independent experiments.

Here, the *serA* deletion mutant (Δ*serA*) of *P. polymyxa* WLY78 was constructed. In the minimal medium without serine, *P. polymyxa* WLY78 grew well, but the Δ*serA* mutant nearly could not grow. The Δ*serA* mutant grew well with the addition of serine in medium and grew best when serine concentration was 1 mM. The data suggested that *serA* is essential for bacterial growth in the absence of serine (Figure 3). The growth of both the Δ*serA* mutant and *P. polymyxa* WLY78 decreased as serine concentration was more than 1 mM.

### 2.3. Characterization of the T-box Riboswitch of the serA Gene

**Figure 4:**
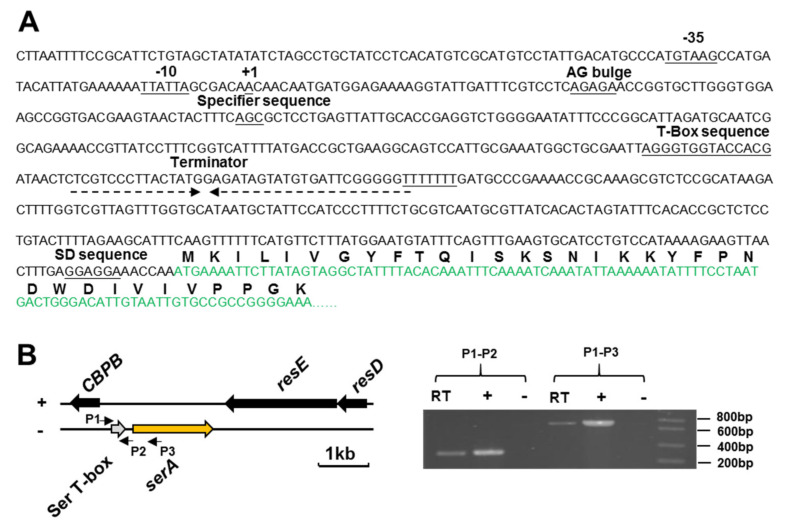
Characterization of the T-box region of the *serA* gene of *P. polymyxa* WLY78. (**A**) The leader sequence and some conserved features of the Ser T-box region. TSS site (+1), −35/−10 region, AG bulge, T-box sequence, and terminator region are shown. (**B**) Identification of Ser T-box RNA by RT-PCR. The Ser T-box region is indicated by an arrow in grey color and *serA* gene in orange color. P1, P2, and P3 that represented by little arrows are primers for RT-PCR. A 252 bp fragment obtained by using P1 and P2 primers and a 686 bp fragment obtained by using P1 and P3 primers are shown on the agarose gel. RT, standard RT-PCR reaction; (–), negative control in which no reverse transcriptase was added to the RT reaction; (+), positive control in which genomic DNA was used as a template in the RT-PCR.

Here, the transcription start site (TSS) of the *serA* gene of *P. polymyxa* WLY78 was determined by using the 5′-RACE (Rapid Amplification of cDNA Ends) method. The TSS of the *serA* gene is located at −508 bp upstream of the translational start site (ATG). A putative promoter (−35 region and −10 region) was identified in front of the TSS (Figure 4A). The data suggested that the mRNA leader region is 508 bp in length. The Ser T-box riboswitch is 280 bp in length from +1 (TSS) to +280 (end of a string of U residues behind the terminator). The conserved motifs: AG bulge, T-box sequence, and rho-independent transcriptional terminator (inverted repeat sequence followed by a string of U residues in the RNA sequence), were identified in the Ser T-box riboswitch.

Reverse Transcription-PCR (RT-PCR) experiments using two pairs of primers (P1 and P2) and (P1 and P3) were performed to determine that the leader region of the *serA* gene was co-transcribed with the *serA* coding region. The primers P1 and P2 were used to amplify a 252 bp of a double cDNA fragment (from +13 bp to +264 bp relative to TSS) that includes T-box RNA. The primers P1 and P3 were designed to amplify a 686 bp of double cDNA fragment from +13 bp to the 190th bp (relative to the translation start site ATG) in the coding region of *serA* that spans across leader sequence and coding region. The data indicated that T-box RNA in the leader RNA of the *serA* gene was co-transcribed with the *serA* coding region (Figure 4B).

### 2.4. The Secondary Structure of Ser T-box RNA

**Figure 5:**
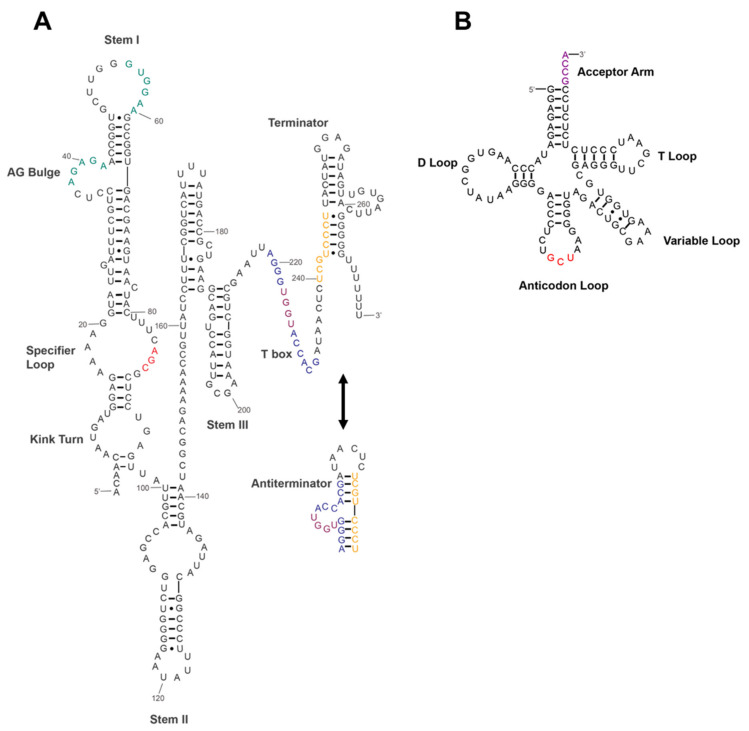
The predicted secondary structures of Ser T-box riboswitch and tRNA^Ser^. (**A**) The Ser T-box sequence is numbered from the transcriptional start site to the end of the transcriptional terminator region. Conserved sequence motifs are labeled in different colors. The specifier sequence (AGC residues complementary to the anticodon sequence of tRNA^Ser^) in the specifier loop is in red. The conserved AG bulge and terminal loop sequences of Stem I are shown in green. The T-box residues (AGGGUGGUACCACG) are shown in blue. The purple-labeled residues (UGGU) within the T-box sequence pair with the acceptor arm sequence of tRNA^Ser^. The orange-labeled residues (UCGUCCCU) interact with the T-box sequence to form an antiterminator structure. (**B**) Cloverleaf structure of tRNA^Ser^. Conserved structure motifs include D-loop, T-loop, Variable loop, and Acceptor arm. The anticodon sequence is shown in red. The sequence (purple) in the acceptor arm base-pair with the Ser T-box antiterminator bulge.

Generally, the T-box RNA should consist of a segment of leader RNA with conserved features that allow recognition of and pairing with the specific charged tRNA^Ser^ or uncharged tRNA. Here, the secondary structure of Ser T-box of *P. polymyxa* WLY78 was predicted by using RNA Folding Form software online (http://unafold.rna.albany.edu/?q=mfold, accessed on 15 September 2020) [36] and the data from T-box Riboswitch Annotation Database (https://tbdb.io, accessed on 25 September 2020) [37]. Ser T-box RNA contains the segments that can form the terminator/antiterminator (Figure 5). In addition, the major structures formed within the T-box RNA include Stem I, Stem II, and Stem III preceding the terminator/antiterminator regions. A kink-turn motif is located at the bottom of Stem I, which modulates the angle of the backbone of the T-box. The specifier loop above the kink-turn motif is responsible for codon-anticodon interaction with seryl-tRNA, and the specifier sequence (AGC), the conserved sequence within the AG bulge (AGAGA) and Stem I terminal loop (GUUGGAA) is able to form a platform to interact with the D-/T-loop of tRNA elbow. Stem II comprises an S-turn motif and a terminal loop. However, Stem II A/B is not found in the 46-nucleotide region between Stem II and III. A 14-nucleotide T-box sequence (5′-AGGGUGGUACCACG-3′) follows Stem III, and this sequence precedes an intrinsic transcription terminator helix in the leader region of the *serA* gene, suggesting a common regulatory mechanism involving transcription attenuation. When intracellular serine concentration is too low, UGGU within the T-box sequence may pair with the acceptor arm of uncharged tRNA^Ser^ to form an antiterminator structure to derepress downstream *serA* expression.

### 2.5. Identification of the Specifier Codon for Ser in the T-box Riboswitch

**Figure 6:**
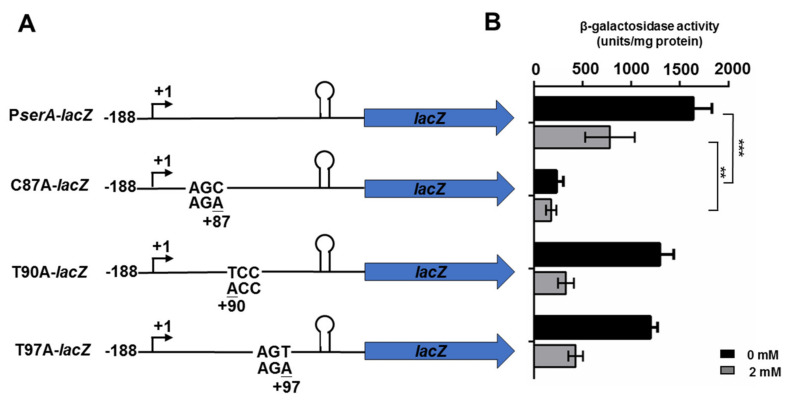
Scheme of mutation of specifier codon of serine and the β-galactosidase activities expressed from the leader-*lacZ* fusion carrying wild-type Ser codon sequence or the mutated codon sequence. (**A**) Scheme of mutation of specifier codon of serine and the leader-*lacZ* fusion carrying wild-type Ser codon sequence or the mutated codon sequence. (**B**) The β-galactosidase activities of *P. polymyxa* strains grown in minimal medium containing 0 and 2 mM L-serine. Results are representative of at least three independent experiments, mean values ± SDs were calculated from three sets of independent experiments. *** *p* < 0.001. ** for *p* < 0.01.

There are three predicted Ser codon sequences: AGC (from +85 to +87 bp relative to transcription start site), UCC (from +90 bp to +92 bp), and AGU (from +95 bp to +97 bp) in the upstream region of the 14-nucleotide T-box sequence (5′-AGGGUGGUACCACG-3′). The three Ser codon sequences AGC, UCC, and AGU were mutated to AGA (glutamate), ACC (histidine), and AGA (glutamate), respectively. A total of 606 bp, including a 188 bp promoter region preceding the TSS and a 508 bp of the leader sequence (containing the wild-type Ser codon sequence or the mutated codon sequence), was fused to the *lacZ* coding region, and the four vectors were transformed into *P. polymyxa* WLY78, yielding P*serA*-lacZ, C87A-*lacZ*, T90A-*lacZ,* and T97A-*lacZ* mutants, respectively. The β-galactosidase activities of these mutants were determined when they were grown in medium containing 2 mM L-serine or without L-serine. As shown in Figure 6, the WT strain had much higher β-galactosidase activity in medium without L-serine than in medium containing 2 mM L-serine. As observed in P*serA*-*lacZ*, T90A-*lacZ,* and T97A-*lacZ* mutants had much higher β-galactosidase activity in medium without L-serine than in medium containing 2 mM L-serine, indicating that mutation of UCC (from +90 bp to +92 bp) or AGU (from +95 bp to +97 bp) did not produce any impacts on regulation exhibited by serine. However, strain C87A-*lacZ* had only the basal β-galactosidase activity in the absence or in the presence of L-serine, suggesting that the mutation of AGC (from +85 bp to +87 bp relative to transcription start site) resulted in the loss of regulation by serine. These results indicated that Ser T-box was a bona fide serine T-box, and the specifier codon of Ser T-box was AGC residues (from +85 to +87 bp relative to transcription start site) that were complementary to the anticodon sequence of tRNA^Ser^. The data suggested that a single codon in the leader region acts as a specifier sequence, controlling the amino acid response.

### 2.6. Mutations of the Conserved Motifs in T-box RNA Affect the serA Expression

**Figure 7:**
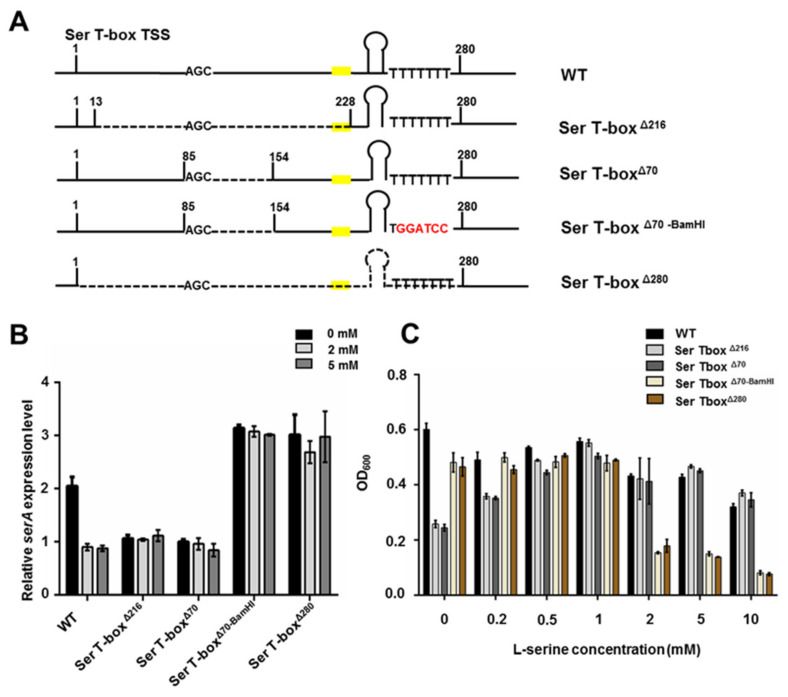
Scheme of mutation of the Ser T-box and the effects on growth and *serA* expression. (**A**) A scheme of mutation of the Ser T-box. The Ser T-box riboswitch is 280 bp in length. The number 1 is the start site of transcription and is also the start site of the Ser T-box riboswitch. The number represents the position of Ser T-box, the dotted line represents the deletion region and red-labeled nucleotides represent the BamHI mutation region. AGC represented the specifier codon and the yellow box represented the position of the 14-bp T-box sequence. (**B**) qRT-PCR analysis of the *serA* expression level of WT, Ser T-box^Δ70^, and Ser T-box^Δ70-BamHI^ strains grown on nitrogen-limited medium with 0, 2, and 5 mM concentrations of L-serine. (**C**) The growth of WT (*P. polymyxa* WLY78) and mutants grown under minimal medium containing 0–5 mM L-serine, and the OD_600_ was measured after 16 h growth within stationary phases. Results are representative of at least three independent experiments, mean values ± SDs were calculated from three sets of independent experiments.

As described above, there are several conserved motifs in Ser T-box RNA of the *serA* gene, including Stem I, Stem II, Stem III, a 14-nucleotide T-box sequence, and terminator/antiterminator helixes. In order to investigate if these motifs are involved in the regulation of *serA* expression, four T-box mutants with the deletion of different lengths of the T-box segments on chromosome were constructed as described in the materials and methods section. As shown in Figure 7A, Ser T-box^Δ216^ was constructed by deleting a 216 bp fragment (from +13 to +228 relative to TSS), including the specific codon AGC for Ser (complementary to the anticodon sequence of tRNA^ser^) and a 14-nucleotide T-box sequence. The Ser T-box^Δ70^ mutant had a deletion of a 70 bp fragment (from +85 to +154 relative to TSS) including the specific codon AGC, but it still contained a 14-nucleotide T-box sequence. The Ser T-box^Δ70-BamHI^ mutant was constructed by changing the sequence TTTTTTT following the terminator to TGGATCC in the Ser T-box^Δ70^ mutant, which was performed by using a restriction site of BamHI (GGATCC) to replace TTTTTT. The Ser T-box^Δ280^ mutant deleted the 280 bp full-length of the Ser T-box riboswitch (from +1 to +280 bp relative to TSS), including TSS, a 14-nucleotide T-box sequence, and the putative terminator sequence.

qRT-PCR analysis revealed that the transcript level of the *serA* gene of *P. polymyxa* WLY78 was much higher in minimal medium without serine than in medium containing 2 or 5 mM serine (Figure 7B), indicating that *serA* expression was inhibited by 2 mM L-serine. However, the expression levels of the *serA* gene in both Ser T-box^Δ216^ and Ser T-box^Δ70^ mutants grown in medium with serine or without serine were continuously low. The transcript levels of both mutants were close to those of the *P. polymyxa* WLY78 in medium containing 2 and 5 mM L-serine. A common feature of Ser T-box^Δ216^ and Ser T-box^Δ70^ mutants is the absence of the specific codon AGC of Ser (complementary to the anticodon sequence of tRNA^ser^). The data suggest that the absence of the specific codon AGC resulted in inhibition of the *serA* expression. Whereas the transcripts of the *serA* in both Ser T-box^Δ70-BamHI^ and Ser T-box^Δ280^ mutants were at similarly high levels whether there was serine or not. And the transcripts of *serA* in both mutants under the two conditions were much higher than that of *P. polymyxa* WLY78 in medium without serine. The data suggest that expression of *serA* in the two mutants was not regulated by the T-box riboswitch according to serine concentration. A common feature of the two mutants is that the terminator structure is destroyed. Thus, deletion of the terminator sequence or mutation of the continuous 7U following the terminator sequence in the two mutants led to transcription through the *serA* coding region.

Effects of serine on the growth of these mutants and *P. polymyxa* WLY78 were determined (Figure 7C). *P. polymyxa* WLY78 grew well in the minimal medium without serine, suggesting that *serA* can synthesize and provide serine for bacterial growth. However, the growth of *P. polymyxa* WLY78 decreased as the serine concentration increased, suggesting that a high concentration of serine inhibited growth and is consistent with the transcription results obtained by qRT-PCR. In contrast, the growth of Ser T-box^Δ216^ and Ser T-box^Δ70^ mutants were obviously lower than that of the wild-type *P. polymyxa* WLY78 in the medium without serine, suggesting synthesis of serine is inhibited and is consistent with the transcription results obtained by qRT-PCR. The growth of both Ser T-box^Δ216^ and Ser T-box^Δ70^ mutants were greatly recovered with the increasing of L-serine concentration, and the highest growth was observed in the presence of 1 mM L-serine and then decreased when L-serine was more than 1 mM. Notably, the growth of both Ser T-box^Δ70-BamHI^ and Ser T-box^Δ280^ mutants was nearly the same high level in the range of 0–1 mM L-serine. However, the growth of both Ser T-box^Δ70-BamHI^ and Ser T-box^Δ280^ mutants was significantly decreased and was much lower than those of the wild-type *P. polymyxa* WLY78 and both Ser T-box^Δ216^ and Ser T-box^Δ70^ mutants when L-serine concentration is more than 2 mM. The data indicate that the growth of both Ser T-box^Δ70-BamHI^ and Ser T-box^Δ280^ mutants was more inhibited by the intracellular serine than other strains. The results are in agreement with the fact that *serA* was constitutively expressed at a high level in both Ser T-box^Δ216^ and Ser T-box^Δ70^ mutants and thus these mutants had a higher amount of the intracellular serine than the wild-type *P. polymyxa* WLY78 and both Ser T-box^Δ216^ and Ser T-box^Δ70^ mutants did.

## 3. Discussion

In this study, the effects of L-serine on growth and nitrogen fixation of *P. polymyxa* WLY78 were studied. We found that L-serine can be used as the sole nitrogen source for the growth of *P. polymyxa* WLY78. This bacterium grew well in the presence of 10 mM L-serine, but the best growth was observed in the presence of 2 mM L-serine, which indicated that too much L-serine inhibited growth. A previous study showed that L-serine would inhibit the growth of *E. coli* cells cultured in minimal medium with some certain carbohydrate as the sole carbon source including glucose by inhibiting homoserine dehydrogenase [33]. In *B. subtilis*, over 1 mM of serine inhibits the growth of cells cultured in minimal medium, while the addition of serine to LB medium does not show any inhibition. Moreover, the addition of threonine partially overcomes serine toxicity, suggesting that the serine toxicity is due to the repression of threonine biosynthesis [38]. It might be the same situation in *P. polymyxa* WLY78.

The nitrogenase activity and *nifH* transcription were inhibited by more than 1 mM L-serine, which was in agreement with that L-serine could be used as the sole nitrogen source. When L-serine was added, the intracellular nitrogen pool elevated and the expression of nitrogen fixation genes was inhibited. Previous studies showed that the effects of amino acids on growth and nitrogen fixation varied greatly among the different *Azospirillum* species. *A. brasilense* grew poorly or not at all on serine as the sole nitrogen and carbon sources, and its nitrogenase activity was inhibited only slightly by even 10 mM serine. In contrast, *A. lipoferum* and *A. amazonense* grew very well on serine as the sole nitrogen and carbon sources, and their nitrogenase activities were severely inhibited by the serine [39]. These results suggested that serine would not inhibit nitrogenase activity when it could not be used as nitrogen source, otherwise, the inhibition on nitrogenase activity existed. The different effects of serine on growth and nitrogen fixation may be due to different regulation mechanisms used by various organisms.

Meanwhile, the lack of serine also inhibits growth and nitrogenase activity, especially for Ser T-box^Δ70^ and Ser T-box^Δ70-BamHI^ mutants, the growth and nitrogenase activities of which decreased obviously without serine addition. *P. polymyxa* WLY78 has a *nif* gene cluster comprising nine genes (*nifB, nifH, nifD, nifK, nifE, nifN, nifX, hesA, and nifV*) encoding nitrogenase [40]. The serine and two downstream amino acids glycine and cysteine in the compositions of the proteins encoded by the nine genes accounted for 6.04%, 8.93%, and 1.93%, respectively. As for all proteins of *P. polymyxa* WLY78, the three amino acids accounted for 6.36%, 7.24%, and 0.77%, respectively, which means more glycine and cysteine demand of nitrogenase compared with the average level, and the two amino acids are synthesized from serine, though the proportion of serine of nine nitrogenase is a little lower than average level (Figure S1). Furthermore, the transcript levels of *P. polymyxa serA* were much higher under nitrogen fixation conditions than in non-nitrogen fixation conditions (Figure S2), suggesting that more serine is synthesized under nitrogen fixation conditions than in non-nitrogen fixation conditions. One possible reason for nitrogenase activities reduction of Ser T-box^Δ70^ and Ser T-box^Δ70-BamHI^ mutants is that the intracellular serine of these two mutants could not meet the demand of the nitrogen fixation process.

Then, we investigated the regulation mechanism of the *serA* expression. A long (508 bp) leader sequence upstream of the *serA* coding region is identified. A T-box riboswitch with several conserved features: a single specifier codon for serine, three stem-loop structures, a 14 bp sequence (the T-box), and an intrinsic transcriptional terminator is found in the long leader region. There are three predicted serine codon sequences: AGC, AGU, and ACC in the leader region. Alternation of each of the three serine codons showed that only the single codon AGC (from +85 to +87 bp relative to TSS) is the specifier codon for serine, consistent with the report that there is only a single specifier sequence complementary to tRNA in the aminoacyl-tRNA synthetase genes of *B. subtilis* [20]. It is predicted that the specifier codon for *B. clausii* and *B. halodurans* is Ser (AGC), while for *Clostridium acetobutylicum* and *Clostridium tetani* is Ser (UCC) [19], and the divergence of specifier codons for different microorganisms is to be researched.

Further, qRT-PCR showed that transcription of the *serA* in the wild-type *P. polymyxa* WLY78 was induced by serine starvation, whereas deletion of the specifier codon (AGC) resulted in nearly no *serA* expression in the presence or absence of serine, suggesting that the specifier codon is essential for the *serA* expression. Deletion of the terminator sequence or the continuous TTTTTTT following the terminator structure led to constitutive expression of *serA,* suggesting that repression exhibited by the terminator is relieved. The data are consistent with the T-box riboswitch regulation system which forms the terminator or antiterminator depending on the aminoacylation state of the bound tRNA in response to amino acid starvation and to control gene expression. Also, the predicated structure of the T-box riboswitch in the leader region of the *serA* in *P. polymyxa* WLY78 has the conserved motifs as found in those of the aminoacyl-tRNA synthetase genes regulated by the T-box mechanism. Thus, our study revealed that the *serA* in *P. polymyxa* WLY78 is regulated by the T-box riboswitch control system in which a tRNA is an effector to modulate gene expression [16]. The predicted secondary structure of the Ser T-box riboswitch (Figure 5) is based on the online database and the published T-box structures. Although structures of Stem I and the terminator region are well-matched, some details still need to be improved on the basis of future experimental evidence.

In addition, we found that a *serC* is located separately from *serA* in *P. polymyxa* WLY78, but there is no T-box region upstream of the *serC* coding region. However, sequence analysis suggests that the *serC* gene in *Clostridium* *thermocellum* and *Desulfitobacterium hafniense* is regulated by a Ser (UCC) T-box RNA [19]. The variations in regulation mechanisms of amino acid synthesis among different organisms reflect that microorganisms take different strategies to control gene expression to adapt to the changing environments. Moreover, *serS* encoding Seryl-tRNA synthetase is a common member of Ser T-box regulon in many Firmicutes including *B. subtilis* and *Paenibacillus* species [34]. A *serB* gene has not been identified in many *Firmicutes* including all *Paenibacillus* species for a long time. A recent study has revealed that *ysaA* encoding haloalkanoate dehalogenase may be the equivalent of *serB* in *B. subtilis*. The *B. subtilis* YsaA showed phosphatase activity against phosphoserine, phosphothreonine, phosphoethanolamine, and histidinol phosphate [41]. We have used the *B. subtilis ysaA* to search the genome of *P. polymyxa* WLY78 and found that there is a *ysaA* gene in this bacterium.

**Figure 8:**
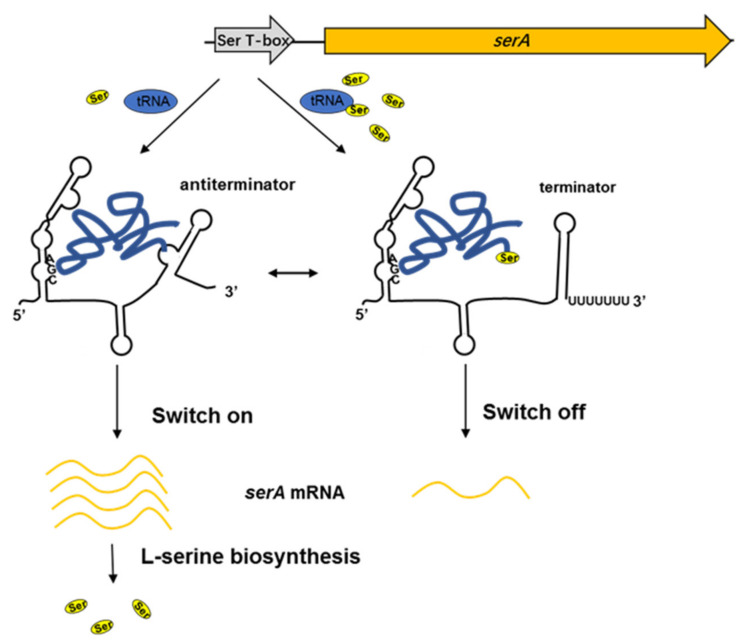
Regulatory model of the Ser T-box riboswitch involved in L-serine biosynthesis in *P. polymyxa* WLY78. In serine starvation, uncharged serine-tRNA pairs with the UGGU bulge within the T-box sequence to form an antiterminator structure, then *serA* expression is switched on. In serine abundance, the charged tRNA can not pair with the 3′ side of the intrinsic terminator region and the *serA* expression is switched off.

According to our results, we proposed a regulatory model of L-serine biosynthesis in *P. polymyxa* WLY78 by the Ser T-box riboswitch (Figure 8). When L-serine is limited, the amount of uncharged serine-tRNA would increase and it pairs with the specifier codons in the T-box region to form an antiterminator, then the *serA* expression is switched on, and eventually, L-serine is synthesized. On the contrary, when intracellular L-serine is in excess, the charged tRNA could not pair with the 3′ side of the terminator region, and then the *serA* expression was shifted to the switch-off state and L-serine biosynthesis was reduced to a relatively low level.

## 4. Materials and Methods

### 4.1. Bacteria Strains, Plasmids, and Growth Conditions

The bacterial strains and plasmids used in this study are shown in Supplementary Table S1. *P. polymyxa* strains were grown in LD medium (per liter contains: 5 g NaCl, 5 g yeast extract, and 10 g tryptone) at 30 °C. For nitrogenase activity assays, *P. polymyxa* strains were grown in a nitrogen-limited medium under anaerobic conditions. Nitrogen-limited medium used in this study contained 10.4 g/L of Na_2_HPO4, 3.4 g/L of KH_2_PO_4_, 26 mg/L of CaCl_2_·2H_2_O, 30 mg/L of MgSO_4_, 0.3 mg/L of MnSO_4_, 36 mg/L of ferric citrate, 7.6 mg Na_2_MoO_4_·2H_2_O, 10 mg/L of p-aminobenzoic acid, 5 mg/L of biotin, and 2% (*w*/*v*) glucose with 2 mM glutamate as the nitrogen source. To measure the growth, strains were grown in a mineral medium that contained 0.5 g/L KH_2_PO_4_, 1.5 g/L Na_2_HPO_4_, 1 g/L NaCl, 0.2 g/L MgSO_4_·7H_2_O, 5 g/L glucose, and 1 g/L NH_4_NO_3_, and different concentrations of L-serine was added in some experiments. When serine was the sole nitrogen source, NH_4_NO_3_ was removed. *Escherichia coli* strains JM109 and DH5α were used as routine cloning hosts. The thermo-sensitive vector pRN5101 was used as a gene cloning vector for gene nutation. The shuttle vector pHY300PLK that can be replicated in *P. polymyxa* strains and *E. coli* was used in the construction of *lacZ* fusion. When appropriate, antibiotics were added in the following concentrations: 100 μg/mL ampicillin, 5 μg/mL erythromycin, and 12.5 μg/mL tetracycline.

### 4.2. Construction of Ser T-box^Δ216^, Ser T-box^Δ70^, Ser T-box^Δ280^, and ΔserA Strains

The in-frame-deletion mutant (Δ*serA*) of the *serA* gene in *P. polymyxa* was constructed. Also, the three mutants (Ser T-box^Δ216^, Ser T-box^Δ70^, Ser T-box^Δ280^) with different length deletion in the leader region of the *serA* gene were constructed by using a homologous recombination method, respectively. For doing this, the upstream fragment (~1 kb) and downstream fragment (~1 kb) flanking the target sequence of Ser T-box and *serA* were amplified by PCR using Phanta® Max Super-Fidelity DNA Polymerase (Vazyme Biotech Co., Ltd., Nanjing, China), respectively. The two fragments were then fused with the HindIII/BamHI digested pRN5101 vector using Gibson assembly master mix (New England Biolabs, USA), generating four recombinant plasmids: pRDSer T-box^Δ216^, pRDSer T-box^Δ70^, pRDSer T-box^Δ280^, and pRD*serA*. The four recombinant plasmids were individually transformed into *P. polymyxa* WLY78 as described by Wang et al. [40]. Then, the single crossover transformants were selected for erythromycin resistance (Em^r^). Subsequently, the double-crossover transformants were selected from the initial erythromycin transformants after several rounds of nonselective growth at 39 °C and confirmed by PCR amplification using corresponding primers and Green Taq Mix (Vazyme Biotech Co., Ltd., Nanjing, China). The primers used here are listed in Supplementary Table S2.

### 4.3. Construction of Ser T-box^Δ70-BamHI^ Strain

The mutant Ser T-box^Δ70-BamHI^ was constructed in the background of the Ser T-box^Δ70^ mutant described above. The final 6T of the continuous 7T sequence (TTTTTT) in the leader region of the *serA* gene were replaced by a restriction site of BamHI (GGATCC) by primer design, the upstream fragment (~1 kb) and downstream fragment (~1 kb) flanking the region containing the continuous T sequences were amplified by PCR from genome DNA of Ser T-box^Δ70^. The two fragments were then fused to the HindIII/BamHI digested pRN5101 vector using Gibson assembly master mix, generating the recombinant plasmid pRUBamHI. The recombinant plasmid was transformed into the Ser T-box^Δ70^ mutant. The single-crossover transformants were selected for erythromycin resistance (Em^r^). Subsequently, the double-crossover transformants were selected from the initial Erythromycin resistance transformants after several rounds of nonselective growth at 39 °C. Then PCR amplification and subsequent digestion with BamHI were used to confirm the mutants. The primers used here are listed in Supplementary Table S2.

### 4.4. Construction of Promoters-lacZ Fusion

A total of 696 bp fragments, including a 188 bp promoter region preceding the TSS (transcription start site) and a 508 bp DNA fragment preceding the of ATG (translation start site) was amplified from the genomic DNA of *P. polymyxa* WLY78 using primers Ser T-boxpU-f and Ser T-boxp-r to construct PSer-*lacZ*. The *lacZ* coding region was amplified from the plasmid pPR9TT with primers lacZ-f and lacZ-r. The site mutation of specific codon was based on PSerA-*lacZ* using primers of MutC87A-f/MutC87A-r, MutT90A-f/MutT90A-r, and MutT97A-f/MutT90A-r by PCR, respectively. The PCR product was digested by DpnI then transformed to JM109 competent cells to select positive mutation transformants by sequencing. The successful site mutation fragment was PCR amplified and assembled to pHY300PLK and then transformed into *P. polymyxa* WLY78.

### 4.5. RNA Preparation, RT-PCR, and qRT-PCR Analysis

The leader region was confirmed by reverse transcription PCR (RT-PCR), and transcription levels of genes were confirmed by quantitative real-time PCR (qRT-PCR). For corresponding experiments, 50 mL of cultures were harvested at 4 °C and rapidly frozen under liquid nitrogen. Total RNAs were extracted with RNAiso Plus (Takara, Japan) according to the manufacturer’s protocol. Synthesis of cDNA was performed using PrimeScript RT reagent Kit with gDNA Eraser (Takara, Japan). For RT-PCR 0.5 μg cDNA was used. qRT-PCR was performed on Applied Biosystems 7500 Real-Time System with the following program: 95 °C for 15 min, 1 cycle; 95 °C for 10 s, and 65 °C for 30 s, 40 cycles. The relative expression level was calculated using the ΔΔCt method, each experiment was performed in triplicate. Primers used for qRT-PCR are listed in Supplementary Table S2.

### 4.6. Acetylene Reduction Assays of Nitrogenase Activity

Acetylene reduction assays were performed as described previously to measure nitrogenase activity [40]. *P. polymyxa* WLY78 and its mutant strains were grown in 5 mL of LD medium overnight. The cultures were collected by centrifugation, washed three times with sterilized water, and then resuspended in a nitrogen-limited medium containing 2 mM glutamate as a nitrogen source to a final OD_600_ of 0.2–0.4. Then, 4 mL of the culture was transferred to a 25-mL test tube and the test tube was sealed with a rubber stopper, L-serine was added to proper concentrations at the same time for special experiments. The headspace in the tube was then evacuated and replaced with argon gas. Then, approximately 2 mL of C_2_H_2_ (10% of the headspace volume) was injected into the test tubes. After incubating the cultures at 30 °C for the corresponding time, a 100 μL gas sample was taken out and injected into gas chromatography to quantify ethylene (C_2_H_4_) production. The nitrogenase activity was expressed in nmol C_2_H_4_/mg protein/hr. All treatments were in three replicates and all the experiments were repeated three or more times.

### 4.7. Identification of Transcription Start Site

The 5′-RACE method was used to determine the transcription start site (TSS) using the SMARTer RACE cDNA Amplification Kit (Clontech, Japan). Gene-specific primers are listed in Supplementary Table S2. The PCR product was cloned into the pGEM^®^-T vector (Promega, 2800 Woods Hollow Road, Madison, WI, USA) after adding A tail by taq DNA polymerase (Takara, Japan) and then sequenced.

## Data Availability

Not applicable.

## Supplementary Materials

The following are available online at https://www.mdpi.com/1422-0067/22/6/3033/s1.
