# Supplementary material for: The Serine Biosynthesis of Paenibacillus polymyxa WLY78 Is Regulated by the T-Box Riboswitch
Source: Int J Mol Sci. 2021 Mar 16;22(6):3033. doi: 10.3390/ijms22063033 (PMC8002221; doi:10.3390/ijms22063033)
Supplement: Supplementary file 1 [file ijms-22-03033-s001.zip › ijms-1139484 suppl proof done/Supplementary Materials/Supplementary Tables.pdf]

Table S1. Strains and plasmids used in this study

Strain/plasmid	Relevant characteristics	Source
	***P. polymyxa* strains**	
WLY78	Wild-type strain	
Δ*serA*	In-frame deletion mutant of the *serA* gene	This study
Ser T-box$^{\Delta 216}$	A mutant of *P. polymyxa* WLY78 having deletion of a 216 bp in the Ser T-box region	This study
Ser T-box$^{\Delta 70}$	A mutant of *P. polymyxa* WLY78 having deletion of a 70 bp in the Ser T-box region	This study
SerT-box$^{\Delta 280}$	A mutant of *P. polymyxa* WLY78 with deletion of the full length of the Ser T-box region (from TSS to polyU)	This study
Ser T-box$^{\Delta 70\text{-BamHI}}$	A mutant of *P. polymyxa* WLY78 with deletion of the full length of the Ser T-box region (from TSS to polyU) with the polyU being replaced by BamHI site	This study
	***E. coli* strains**	
JM109	General cloning host; recA1, endA1, gyrA96, thi-1, hsdR17, supE44, relA1, Δ(lac-proAB)/F′ [traD36, proAB+, lacIq, lacZΔM15]	Sangon Biotech Co.
	**Plasmids**	
pRN5101	Temperature-sensitive *E. coli* - *Bacillus* shuttle vector, Em^r	
pHY300PLK	Multiple-copy *E. coli* - *Bacillus* shuttle vector, Tc^r	Takara
pPR9TT	Broad-host range *lacZ* promoter probe vector; RK2 replicon; Amp^r, Cm^r	Sangon Biotech Co.
pRDserA	A derivative of pRN5101 vector for construction of the *serA* deletion mutant	This study
pRDSer T-box$^{\Delta 216}$	A derivative of pRN5101 vector for construction of the Ser T-box$^{\Delta 216}$ mutant	This study
pRDSer T-box$^{\Delta 70}$	A derivative of pRN5101 vector for construction of the Ser T-box$^{\Delta 70}$ mutant	This study
pRDSer T-box$^{\Delta 280}$	A derivative of pRN5101 vector for construction of the Ser T-box$^{\Delta 280}$ mutant	This study
pRUBamHI	A derivative of pRN5101 vector for construction of the Ser Ser T-box$^{\Delta 70\text{-BamHI}}$ mutant in which polyU was replaced by BamHI	This study
	***lacZ* fusion**	
pSerA-lacZ	A derivative of pHY300PLK vector carrying promoter and leader region of the *serA* fused to the *lacZ* coding region from pPR9TT vector	This study
pSI-lacZ	A derivative of pHY300PLK vector carrying the mutated leader region (AGC being mutated to AGA) fused to the *lacZ* coding region from pPR9TT vector	This study
pSII-lacZ	A derivative of pHY300PLK vector carrying the mutated leader region (TCC being mutated to ACC) fused to the *lacZ* coding region	This study
pSIII-lacZ	A derivative of pHY300PLK vector carrying the mutated leader region (AGT being mutated to AGA) fused to the *lacZ* coding region	This study

Table S2. Primers used in this study

Primer Name	Sequence (5′→3′)	PCR product
SerTBupF	acgatgcgtccggcgtagagTCATCAATGGCTTCAACAAG	Upstream region of Tbox$^{\Delta 216}$
SerTBupR	aacaatgatgGATAACTCTCGTCCCTTAC	
SerTBdnF	gagagttatcCATCATTGTTGTTGTCGC	Downstream region of Tbox$^{\Delta 216}$
SerTBdnR	cgcaaaagacataatcgataCTCAAAAAATTTAAAAACCACTTG	
SerTB70upR	aactactttcGTTATCCTTTCGGTCATTTTATG	Upstream region of Tbox$^{\Delta 70}$
SerTB70dnF	aaaggataacGAAAGTAGTTACTTCGTCAC	Downstream region of Tbox$^{\Delta 70}$
serAupF	acgatgcgtccggcgtagaggatccAAGCTAGAGAAGATGCGG	Upstream region of *serA*
serAupR	aacacaacgcCATTTCCTGCTCATCTCG	
serAdnF	gcaggaaatgGCGTTGTGTTGCTGCTTC	Downstream region of *serA*
serAdnR	cgcaaaagacataatcgataAAAATATCCAGATTGAGCCAATCAG	
SerTB280upR	aacaatgatgGATGCCCGAAAACCGCAAAG	Upstream region of Tbox$^{\Delta 280}$

SerTB280dnF	ttcgggcatcCATCATTGTTGTTGTCGC	Downstream region of Tbox$^{\Delta 280}$
SerTBbamHIupR	GGGGGTGGATCCGATGCCCGAAAACCGCAAA	Upstream region of Ser T-box$^{\Delta 70\text{-BamHI}}$
SerTBbamHIdnF	GGCATCGGATCCACCCCCGAATCACATACTA	Downstream region of Ser T-box$^{\Delta 70\text{-BamHI}}$
	***lacZ* fusion**	
Ser T-boxp-f	tagagatctgcaggtcgacgAGTTAGTTAGTCCTCTGC	Ser T-box promoter cloning
Ser T-boxp-r	gtaaaacgacCATGTTACTTCGTCACCG	
lacZ-f	atgGTCGTTTTACAACGTCGTG	*lacZ* cloning
lacZ-r	ttataacaggaattcccgggTTATTTTTGACACCAGACC	
MutC87A-f	TAACTACTTTCAGAGCTCC	AGA mutation
MutC87A-r	AGTAGTTACTTCGTCACC	
MutT90A-f	AACTACTTTCAGCGCACCTG	TCC mutation
MutT90A-r	AGTAGTTACTTCGTCACCGG	
MutT97A-f	AGCGCTCCTGAGATATTG	AGT mutation
MutT97A-r	GGAGCGCTGAAAGTAGTT	
	**For RT-PCR**	
RTserA-r	TACCTGCCGTCATGATGC	T-box verification
RTser Tbox-f	GAGAAAAGGTATTGATTTCGTCC	
RTser Tbox-r	CACATACTATCTCCATAGT	
	**For qRT-PCR**	
q16s-f	TTTGTCGTCAGCCTCGTGTTCGTG	16S rDNA
q16s-r	ATCCCCACCTTCCTCCGGTTTG	
qserA-f	GCTGGCAGGCTATGGCGAAC	*serA*
qserA-r	GGTGCCGACACGACCGATAATG	
qnifH-f	AACAGCCGGAATACGGACC	*nifH*
qnifH-r	ACCTGCCAGCTCTTCATACTC	
