# Supplementary figures and images for: The Serine Biosynthesis of Paenibacillus polymyxa WLY78 Is Regulated by the T-Box Riboswitch
Source: Int J Mol Sci. 2021 Mar 16;22(6):3033. doi: 10.3390/ijms22063033 (PMC8002221; doi:10.3390/ijms22063033)

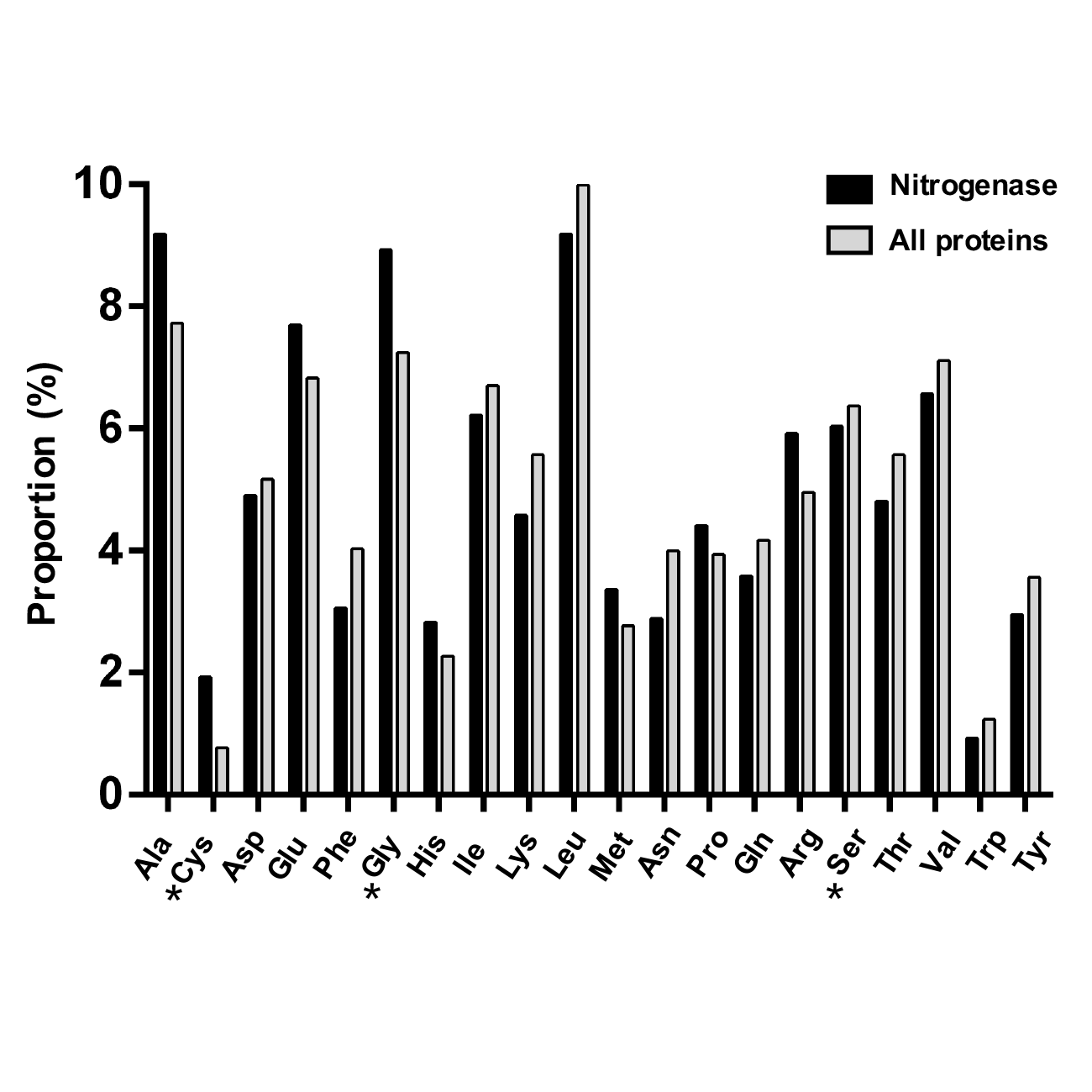

Supplement: Supplementary file 1 [file ijms-22-03033-s001.zip › ijms-1139484 suppl proof done/Supplementary Materials/figure S1.tif]

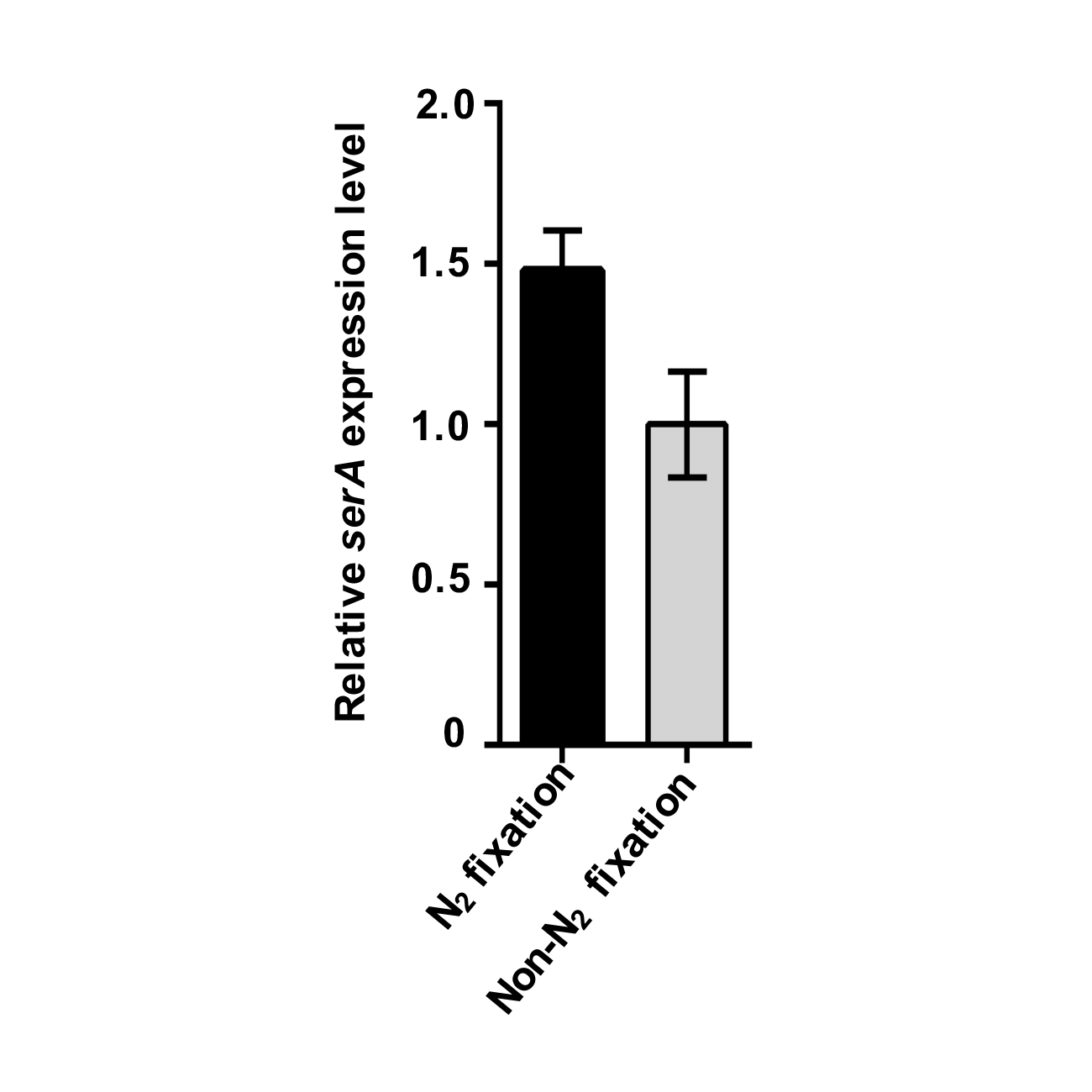

Supplement: Supplementary file 1 [file ijms-22-03033-s001.zip › ijms-1139484 suppl proof done/Supplementary Materials/figure S2.tif]
